# Salmonella Meningitis in a Young Infant: A Case Report

**DOI:** 10.7759/cureus.44147

**Published:** 2023-08-26

**Authors:** Aziza Elouali, Nourelhouda Ouerradi, Ghanam Ayad, Abdeladim Babakhouya, Maria Rkain

**Affiliations:** 1 Department of Pediatrics, University Hospital Mohammed VI, Faculty of Medicine and Pharmacy, Mohammed First University, Oujda, MAR

**Keywords:** meningitis, case report, infant, children, salmonella

## Abstract

Salmonella meningitis is a rare but severe form of bacterial meningitis. It is most frequently diagnosed in infants under one year of age, especially those under the age of three months, from emerging and underdeveloped countries and with a tropical climate. Salmonella meningitis has been associated with a high mortality rate, as well as a high risk of relapse and significant neurological complications such as cerebral palsy, visual and hearing impairments, and mental retardation. The treatment for Salmonella meningitis is challenging, and there is no consensus on the best approach. In this report, we describe a case of Salmonella meningitis in a 37-day-old girl exclusively formula-fed baby girl who was admitted for high fever, irritability, poor feeding, low activity, excessive crying for five days, and repeated seizures on the day of admission. The infant was treated with a four-week course of intravenous antibiotics. Given the severity of this infection and its potential long-term consequences, early diagnosis, and prompt treatment are crucial. However, this patient recovered without neurological disorders.

## Introduction

Salmonella meningitis (SM) is a rare but severe form of bacterial meningitis, due to Salmonella bacteria, the Gram-negative, facultatively anaerobic flagellate bacilli, found in contaminated food. SM is accounting for less than 1% of cases in developed countries. This rate may occur in up to 13% of developing countries. Its treatment is difficult and not consensual [1]. Previous studies have indicated that Salmonella meningitis is often accompanied by a high incidence of morbidity, with varying complications (ranging from 50-90%), and a mortality rate reaching up to 50-70% [2]. In this report, we present the case of meningitis in a 37-day-old infant who was successfully treated with a prolonged course of intravenous antibiotics. This case evidences difficulty using an appropriate antibiotic regimen due to the lack of guidelines and that the infant was successfully treated with imipenem and ciprofloxacin.

## Case presentation

We present the case of a 37-day-old female infant who was admitted to the Emergency Department in a Tertiary Care Hospital for focal clonic seizures involving the right arm with facial involvement, that lasted for 10-15 minutes. The patient was born at full term by ceasarean section with a birth weight of 3500g, a head circumference of 35cm, and an Apgar score of 10. She was bottle-fed. The parents were not blood-related, and the infant had an older sister. At birth, the patient received Hepatitis B and Bacillus Calmette-Guérin (BCG) vaccines on day 7. The infant presented with a five-day history of high fever (39.5-40 °C), irritability, poor feeding, lethargy, and excessive crying. The day after the onset of symptoms, the baby was examined by a private pediatrician. She was treated with ceftriaxone (ceftriaxone 50 mg/kg/day intramuscularly). After three days of antibiotics, the infant evolved with gradual prostration, irritability, persistent fever, and focal clonic seizures. The patient was then referred to a tertiary care hospital for further management. Upon admission, the infant was lethargic with a pulse rate of 154 beats per minute, respiratory rate of 40 per minute, blood pressure of 100/60 mm Hg, capillary refill of two seconds, and temperature of 39.5°C. Physical examination revealed a bulging anterior fontanelle with no neck stiffness and a head circumference of 38 cm. Neurological examination revealed incomplete Moro reflex and poor suck reflex. Examination of the chest, heart, and abdomen revealed no abnormalities. The diagnosis of bacterial meningitis was strongly suspected based on the physical exam findings, hence, a cerebrospinal fluid (CSF) analysis and Cranial Computed Tomography (CT) scan and blood culture were ordered. Then the infant was started empirically on intravenous antibiotics (ceftriaxone intravenous {IV} 100mg/Kg/day and gentamicin 3mg/Kg/day), antiepileptics (phenobarbitone loading dose: 20mg/kg over 20 minutes, and then a maintenance dose of 5 mg/kg per day), IV fluids, and symptomatic treatment. The cranial CT scan reported normal findings. The CSF analysis results are given in Table 1.

**Table 1:** Analysis of peripheral blood count and CSF at the time of diagnosis CSF: Cerebrospinal fluid

Peripheral blood data	
Total WBC/ µl	9700
Platelets *10^4^/ µl	20.8
Hemoglobin level g/dl	10.9
CSF data	
Color	Xanthochromic
Total WBC/ µl	260
Glucose (mg/dl)	6
Protein (g/l)	3.34
CSF/blood glucose ratio	<0.5
Gram Strain negative	+

**Figure 1:**
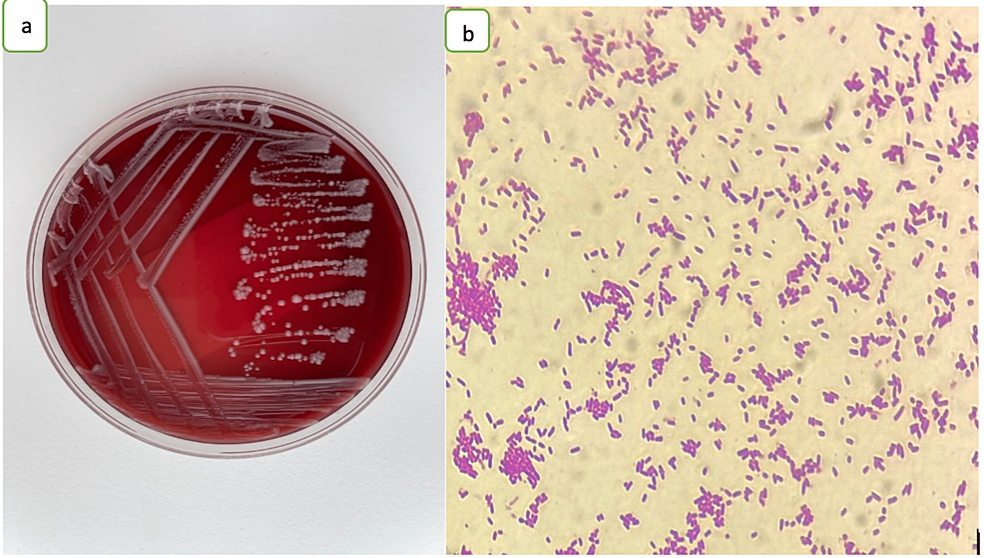
Histopathology study (a) Morphology and Cultural Aspects of Salmonella spp.  (b) Colonies are opaque, translucent, or transparent, and generally with a black center.

The initial blood investigations showed a C-reactive protein level of 310 mg/l, the serum electrolytes, liver function, and renal function tests were normal. On the second day of hospitalization, the CSF culture showed Gram-negative bacilli, later identified as Salmonella spp. (Figure 1) sensitive to ceftriaxone, ampicillin, ciprofloxacin, cotrimoxazole, meropenem and imipenem and resistant to amikacin, gentamicin and tobramycin.

**Figure 2:**
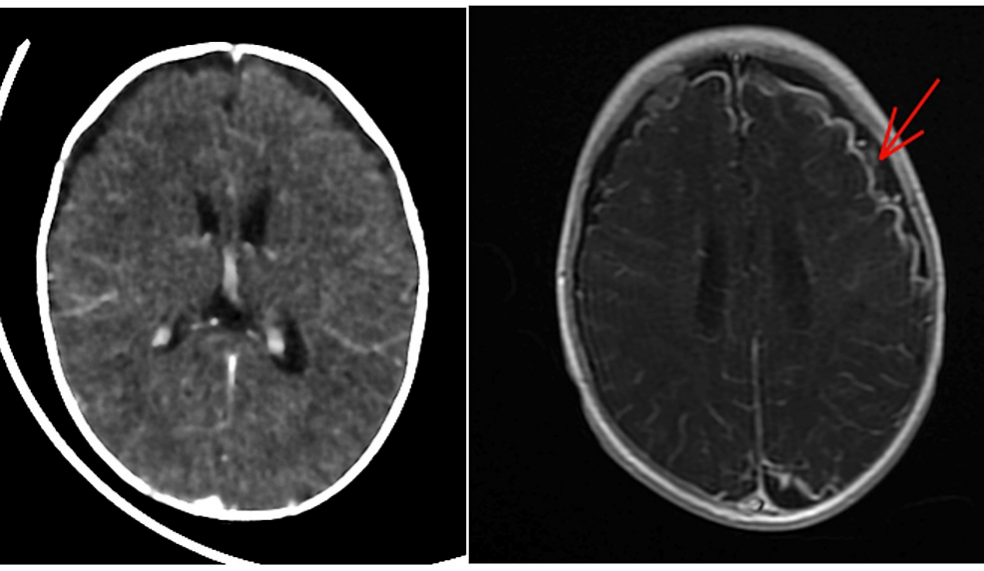
MRI of the brain Brain MRI showed leptomeningeal enhancement and minimal quadri-ventricular hydrocephalus (red arrow).

Due to persisting fever after 72 h of ceftriaxone and amikacin therapy, the child was started on IV ceftriaxone and ciprofloxacin. After five days of antibiotic treatment, the infant maintained feverish peaks and frequent focal tonic seizures daily. Repeat investigations revealed an increased CRP, total leukocyte count, and thrombocytosis. The blood count showed: hemoglobin 8.3 g/dL, WBC 21800/mm^3^ (segmented neutrophils 55.0%, lymphocytes 27.0%, monocytes 16.0%, eosinophils 0.59%), platelets 802,000/mm^3^, CRP 338mg/l. However, repeat blood and cerebrospinal fluid cultures and sensitivity tests were negative. An MRI of the brain (Figure 2) done on day 13 showed leptomeningeal enhancement and minimal quadri-ventricular hydrocephalus, and surgical intervention was not advised.

After repeated investigations, and due to no response, the infant's antibiotics were changed to imipenem and ciprofloxacin. Levetiracetam was added for refractory seizures. After 10 days of combined antibiotic treatment that included ciprofloxacin and imipenem, the infant made a full recovery. To identify a possible source of infection, the infant’s mother was also examined. Blood culture and stool cultures were performed but no pathogen was detected. The stool cultures of the father, and sister were also negative. In the evaluation performed for possible underlying immunodeficiencies, immunoglobulin levels, lymphocyte panel and were determined normal. The patient is still being followed up regularly and neurologic examination is all normal. The child receives routine consultations and, at 13 months old, displays appropriate psychomotor and growth development without any neurological sequelae.

## Discussion

Bacterial meningitis remains the leading cause of morbidity and mortality in newborns and children worldwide. Suspected cases of bacterial meningitis are medical emergencies requiring immediate action to establish a specific diagnosis and prompt empiric antimicrobial therapy initiation [3]. Group B Streptococcus, Staphylococcus aureus, Enterococcus, and other Gram-negative bacilli (e.g., Enterobacter) are the most common organisms that cause meningitis in newborns and young children [4]. Meningitis caused by Salmonella is a relatively rare disease. In 1907, Ghon identified the first case of Salmonella meningitis. Since then, there have been few relevant publications on this topic, mostly limited to single cases or small case series, indicating the rare occurrence of this pathology [3]. Salmonella meningitis in acute bacterial meningitis has been rarely reported (1%) in developed countries. However, in Africa, Salmonella spp. account for 13% of cases of childhood bacterial meningitis [5]. Immunosuppression, whether congenital or acquired undeveloped blood-brain barrier children, significantly those are risk factors for developing invasive nontyphoidal Salmonella (NTS) infections [6]. Immunodeficiency testing was performed on our patient, but the results were negative. The treatment of Salmonella meningitis is challenging and has never been standardized [7]. Because Salmonella is a facultative intracellular organism, drug penetration may be insufficient, leading to infection progression. In addition, there is growing evidence that resistance to commonly used antibiotics such as ampicillin, chloramphenicol, co-trimoxazole, and cephalosporins is increasing. Treatment regimens for Salmonella meningitis must be adjusted according to the organism's susceptibility pattern and clinical response to the antibiotics used. Recent studies now recommend initiating third-generation cephalosporins (C3G) and ciprofloxacin as first-line therapy [8]. The same regimen was followed in our case after receiving the results of the CSF culture but there was no sign of improvement. Carbapenems may be considered a treatment option, especially in patients who do not respond to initial therapy or relapse [1]. Our patient maintained feverish peaks and focal tonic-clonic seizures. For this reason, imipenem antibiotic treatment was added to ciprofloxacin and the combination was given for four weeks. The duration of the treatment ranged from two to eight weeks [9]. Because of the risk of recurrence, neuroimaging is recommended for all patients with Salmonella meningitis, even if the clinical course is satisfactory [10]. In our patient, transfontanellar ultrasound was requested regularly, and an MRI of the brain was performed, which showed leptomeningeal enhancement and minimal quadri-ventricular hydrocephalus. The prognosis for Salmonella meningitis is generally poor, especially among neonates and infants. Many patients either die or survive with complications such as prolonged seizures, hydrocephalus, subdural collections, cerebral infarctions, ventriculitis, empyema, intracranial abscesses, and cranial nerve palsies. Because of the risk of recurrence, neuroimaging is recommended for all patients with Salmonella meningitis, even if the clinical course is satisfactory [7]. Long-term neurologic sequelae can include speech impairment, movement impairment, intellectual disability, epilepsy, sensorineural hearing loss, visual impairment, abductor paralysis, microcephaly, and hydrocephalus [7]. During the follow-up, the patient had no recurrence of meningitis or neurological sequelae. However, long-term follow-up for neurodevelopment is recommended.

## Conclusions

Salmonella meningitis is a severe disease associated with high mortality and morbidity rates. Patients with Salmonella meningitis require monitoring for acute complications, as well as long-term neurological sequelae. There is a need for more model experts and studies that provide recommendations on treating Salmonella meningitis as currently, all available regimens have been only reported on a case-by-case basis, providing no consensus on an acceptable treatment modality. Carbapenems may be considered a choice in the treatment of meningitis in relapsed cases or where there seems to be no clinical response to third-generation cephalosporines or ciprofloxacin.
